# Recommendations for optimizing the use of dysmorphic red blood cell counts in the clinical laboratory

**DOI:** 10.1515/almed-2026-0048

**Published:** 2026-06-30

**Authors:** Oscar D. Pons-Belda, María José Ferri, Alejandro Almería-Lafuente, Cristina Izquierdo-Álvarez, Sergio Luis-Lima, Miriam Menacho-Román, Mario Román-Cabezas, Marta Sáenz-Valls, Elena Togores-Pérez, Patricia Fernández-Riejos

**Affiliations:** Department of Clinical Laboratory, Hospital Can Misses, Eivissa, Spain; Department of Clinical Laboratory, Hospital General Universitario Dr. Balmis, Alicante, Spain; Department of Clinical Laboratory, Hospital Universitario Miguel Servet, Zaragoza, Spain; Department of Clinical Laboratory, Hospital Universitario Puerta de Hierro, Majadahonda, Spain; Department of Clinical Laboratory, Complejo Hospitalario Universitario de Canarias, Tenerife, Spain; Department of Clinical Laboratory, Hospital Universitario Ramón y Cajal, Madrid, Spain; Department of Clinical Laboratory, Hospital Universitario Virgen del Rocio, Sevilla, Spain; Department of Clinical Laboratory, Hospital HM Modelo, A Coruña, Spain; Department of Clinical Laboratory, Hospital Universitario Virgen de la Macarena, Sevilla, Spain

**Keywords:** dysmorphic red blood cells, acanthocytes, urinary sediment, haematuria

## Abstract

The assessment of dysmorphic red blood cells (RBCs) is of significant clinical interest in the evaluation of haematuria, particularly as a key tool for suggesting a possible glomerular origin. However, its actual utility depends on cautious, context-based interpretation supported by well-defined technical requirements. The lack of universal standardization, interobserver variability, and the influence of preanalytical factors continue to contribute to the heterogeneous implementation of this test in clinical practice. This document provides recommendations aimed at optimizing its use in the clinical laboratory. It reviews the underlying pathophysiological basis, principal indications, and the preanalytical and analytical conditions required to ensure reliable evaluation, as well as its integration with other findings from urinalysis and renal biochemistry. In this regard, erythrocyte dysmorphism should not be considered an isolated test, but rather a complementary parameter whose diagnostic performance improves when interpreted alongside proteinuria or albuminuria, renal function, the presence of pathological casts, and the patient’s clinical context. Furthermore, the importance of a selective approach is emphasized, prioritizing clinical scenarios with higher diagnostic yield, such as persistent microhaematuria, haematuria associated with proteinuria, or suspected glomerular disease. Finally, guidance criteria are proposed for reporting and for integration into multidisciplinary clinical algorithms. Overall, these recommendations aim to promote a more rational, harmonized, and clinically meaningful use of dysmorphic RBC counts within advanced urinalysis.

## Introduction

Haematuria is a common clinical and laboratory finding that may represent the first manifestation of abnormalities located anywhere along the urinary tract, from the glomerulus to the lower urinary tract. Its clinical relevance lies in its association with both benign, transient conditions and more severe renal or urological diseases; therefore, its accurate identification and interpretation constitute an essential step in the initial evaluation of the patient [1].

From a laboratory perspective, urine dipstick testing is widely used as an initial screening method due to its high sensitivity for detecting the pseudoperoxidase activity of haemoglobin. However, the diagnosis of haematuria requires confirmation of erythrocytes by microscopic examination of the urinary sediment, as several conditions may lead to misleading results when dipstick testing is used as a standalone screening tool [2]. False-positive results may arise from haemoglobinuria and myoglobinuria, as well as menstrual contamination, the presence of oxidizing agents such as sodium hypochlorite, bacterial peroxidase activity, or even the consumption of certain vegetables. Conversely, false-negative results are mainly associated with high concentrations of ascorbic acid and with specific physicochemical characteristics of urine, such as high specific gravity or markedly acidic pH [1], 2].

Traditionally, haematuria is classified as macroscopic or microscopic. Macroscopic haematuria is characterized by an abnormal coloration of urine visible to the naked eye and is typically associated with processes affecting the lower urinary tract or the collecting system, such as urinary tract infections, urolithiasis, trauma, or urothelial neoplasms. In contrast, microscopic haematuria does not necessarily alter the visual appearance of urine and is detected only through laboratory testing. When persistent and accompanied by proteinuria, it acquires particular relevance as a marker of renal parenchymal disease, especially in the context of glomerulopathies and systemic disorders [1], 3].

The evaluation of dysmorphic red blood cells (RBCs) has become an established tool to support the differentiation between glomerular and non-glomerular (urological) haematuria [2], 4]. Its pathophysiological basis lies in the fact that, during their passage through a damaged glomerular filtration barrier and along the renal tubules, erythrocytes undergo mechanical, osmotic, and pH-related stress, leading to morphological alterations. In contrast, in postglomerular haematuria, erythrocytes typically retain a more homogeneous morphology, closely resembling that of circulating erythrocytes [5], 6].

Despite its clinical relevance, the assessment of dysmorphic RBCs still faces important limitations, including methodological heterogeneity, lack of universal standardization of morphological criteria, interobserver variability, and the influence of preanalytical and analytical factors [2], 4]. These issues have led to uneven implementation in routine clinical practice and have hindered its consistent integration into diagnostic algorithms in the clinical laboratory.

Accordingly, there is a clear need to establish recommendations aimed at optimizing the use of dysmorphic RBC counts, clearly defining their indications, technical requirements, interpretative limitations, and integration into laboratory reports. This document proposes a consensus framework to promote a rational, reproducible, and clinically meaningful use of this determination.

## Objectives

The objectives of this document are: (a) to define the clinical relevance of dysmorphic RBC counts in the clinical laboratory; (b) to establish recommendations for their rational use; (c) to propose preanalytical and analytical standardization criteria; (d) to promote harmonized interpretation; and (e) to facilitate their integration into multidisciplinary clinical pathways involving the laboratory, nephrology, primary care, and urology.

## Pathophysiological basis

Dysmorphic RBCs are defined as erythrocytes that have lost their typical biconcave morphology. Their detection in urine is generally associated with a glomerular origin of haematuria, as they derive from erythrocytes that have passed through a structurally altered glomerular filtration barrier. During this process, RBCs undergo significant mechanical deformation while passing through a highly restrictive anatomical filter composed of fenestrated endothelium, the glomerular basement membrane, and podocytes [2], [4], [5], [6].

Once within the renal tubule, erythrocytes are subjected to further modifications induced by the urinary environment, particularly variations in tonicity and pH. These conditions promote alterations in membrane proteins, intracellular calcium influx, and cytoskeletal disorganization, thereby contributing to the progressive loss of their original morphology. In addition, dysmorphic erythrocytes have been reported to exhibit lower haemoglobin content compared to isomorphic RBCs [4].

**Figure 1:**
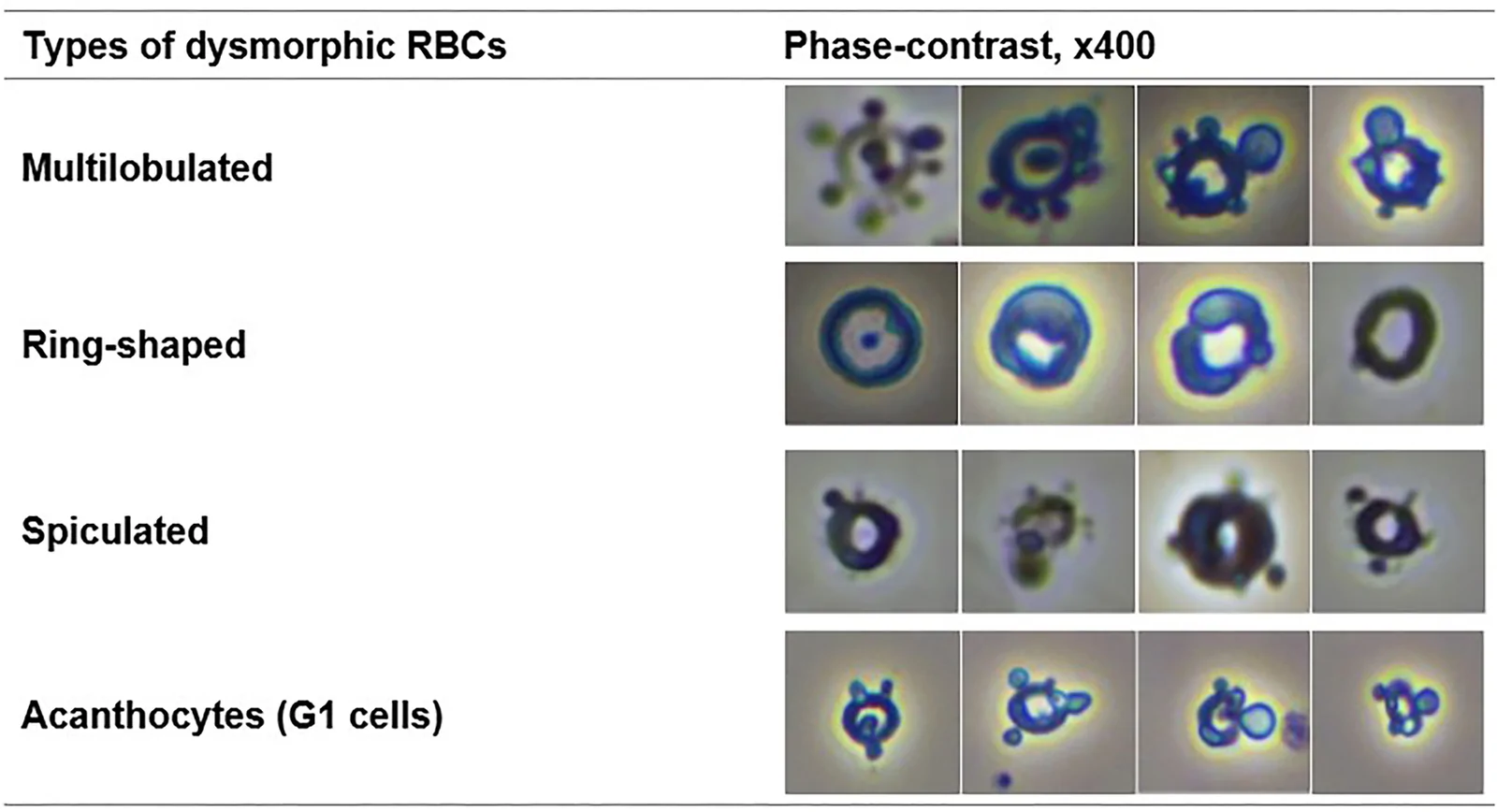
Representative microscopy images of urinary dysmorphic red blood cells (phase-contrast, ×400).

From a morphological perspective, dysmorphic RBCs display marked heterogeneity and may appear under microscopy as multilobulated, ring-shaped, spiculated, or mixed forms [5]. Among these, acanthocytes, also referred to as G1 cells (ring-shaped erythrocytes with irregular vesicle-like protrusions), represent the most characteristic and suggestive morphological variant of glomerular haematuria [2], [4], [5], [6], [7]. Representative microscopy images of the main dysmorphic RBCs are provided in Figure 1.

However, the association between erythrocyte dysmorphism and glomerular disease is not absolute. The presence of dysmorphic RBCs has also been documented in the absence of evident glomerular pathology, underscoring the importance of establishing appropriate diagnostic cut-off values [4].

## Clinical scope and diagnostic utility

The main clinical value of dysmorphic RBC counting lies in its ability to aid in distinguishing between glomerular and non-glomerular haematuria [2], [3], [4], [5, 7]. This distinction is particularly relevant in specific clinical scenarios, including patients with persistent microhaematuria, haematuria associated with proteinuria, suspected renal parenchymal disease, isolated haematuria in young individuals, the concomitant presence of erythrocyte casts or other findings suggestive of glomerular injury, as well as in the follow-up of patients with known or suspected glomerular nephropathies.

However, the diagnostic utility of this parameter is maximized when its interpretation is integrated with other analytical and clinical data [2]. Key complementary parameters include albuminuria or proteinuria, serum creatinine levels and estimated glomerular filtration rate, the presence of urinary casts (particularly erythrocyte casts), leukocyturia not attributable to infection, and the patient’s clinical and immunological context.

Within this framework, dysmorphic RBC counts may serve as a useful adjunctive tool for prioritizing referral to nephrology. Nevertheless, their value should always be considered complementary, as they do not replace comprehensive clinical assessment, quantification of proteinuria, or the performance of other specific diagnostic tests.

## Recommended indications

The assessment of dysmorphic RBCs should not be performed indiscriminately in all urine samples presenting with haematuria. A selective strategy is recommended, based on well-defined clinical and analytical indications. These indications are summarized in Table 1 [1], [2], [3], [4], [5].

**Table 1:** Indications for the assessment of dysmorphic RBCs in urine [1], [2], [3], [4], [5].

Indication	Clinical scenario
Priority (high diagnostic yield)	Persistent microhaematuria confirmed in repeated samples.
	Haematuria associated with proteinuria or albuminuria.
	Haematuria accompanied by impaired renal function.
	Clinical suspicion of glomerulonephritis or glomerular kidney disease.
	Haematuria in paediatric patients or young adults, especially with a family history of kidney disease.
	Haematuria with concomitant erythrocyte casts or other pathological casts.
	Follow-up of patients with IgA nephropathy, Alport syndrome, thin basement membrane disease, or other glomerulopathies.

Non-priority (low diagnostic yield)	Overt macroscopic haematuria.
	Clear clinical suspicion of urological origin (urolithiasis, tumour, instrumentation, confirmed urinary tract infection).
	Aged urine samples.
	Patients with menstruation or evident genital contamination.

## Preanalytical requirements

The preanalytical phase is a critical component in ensuring the reliability of erythrocyte morphological assessment. In this regard, it is advisable for laboratories to implement standardized operating procedures and clearly defined sample acceptance criteria in order to minimize variability and reduce the risk of misinterpretation.

Regarding sample type, the preferred specimen for dysmorphic RBCs assessment is urine from the second morning void, collected as a midstream sample within 2 h of the previous urination [2]. When this condition cannot be met, a random midstream urine sample may be considered an acceptable alternative. In all cases, collection should follow appropriate hygienic measures and midstream technique.

Time to analysis represents another key factor. Samples should be processed as soon as possible, preferably within 2 h of voiding. Delays in processing promote lysis, crenation, and the development of morphological artifacts that may compromise the accurate identification of dysmorphic RBCs. If immediate analysis is not feasible, storage conditions should be previously validated by each laboratory. It should be noted that refrigeration, although useful for preserving overall sample stability, may partially alter formed elements and promote urinary crystallization [2], [3], [4], [5]. In practical terms, validation of refrigerated storage should include, at minimum, assessment of the maximum acceptable storage time at 4–8 °C, comparison with fresh samples analysed within the recommended time window, evaluation of total RBC recovery, preservation of dysmorphic RBC and acanthocyte morphology, and the potential appearance of artefactual changes such as crenation, lysis, or increased precipitation of urates and phosphates. If crystallization or precipitates interfere with microscopic interpretation, a new specimen should be requested and, when feasible, examined at room temperature under controlled conditions. Laboratories should document the locally validated storage interval and include a cautionary comment when delayed analysis may compromise the reliability of erythrocyte morphology assessment.

Additionally, there are specific situations in which dysmorphic RBC assessment should be avoided or, at least, interpreted with caution. These include aged samples, menstrual contamination, and specimens with abundant mucus, crystals, or debris that significantly hinder microscopic evaluation. Similarly, caution is warranted when analyzing urine samples obtained after intense physical exercise, trauma, urological instrumentation, or recent catheterization, particularly in the absence of sufficient clinical information to properly contextualize the findings [1], 3], 4].

Finally, to optimize result interpretation, it is highly desirable that the laboratory has access to relevant minimal clinical information whenever possible. This should include the reason for the test request, persistence or recurrence of haematuria over time, the presence of proteinuria or albuminuria, clinical suspicion of nephrological or urological disease, concomitant urinary tract infection, use of anticoagulant therapy, and history of urolithiasis, urological malignancy, or glomerular disease [1], 3]. The availability of such information plays a decisive role in achieving a more accurate, contextualized, and clinically meaningful interpretation of dysmorphic RBC counts.

## Analytical requirements

Whenever possible, the assessment of dysmorphic RBCs should be performed in uncentrifuged urine, as centrifugation may alter the relative proportion of dysmorphic forms and lead to selective loss, particularly of those with lower density. However, in routine clinical practice, this approach is not always feasible. In such cases, centrifugation followed by sample concentration may be used as an alternative [2]. When centrifugation is required to increase particle concentration, low-speed centrifugation should be used in order to minimize cellular distortion and loss. A practical standardized approach is centrifugation at approximately 400×*g* for 5 min, followed by gentle resuspension of the sediment in a defined residual volume. Laboratories adopting centrifuged sediment for dysmorphic RBC assessment should verify RBC recovery by comparing post-centrifugation counts with the original RBC concentration in uncentrifuged urine and should apply the same procedure consistently in routine practice.

Phase-contrast microscopy is considered the gold standard for the detection and differentiation of urinary particles and represents the technique of choice for erythrocyte morphological evaluation [2], 5], 6]. Laboratories that do not have access to this technology should explicitly acknowledge this limitation, as it may affect both diagnostic sensitivity and reproducibility. In this context, bright-field microscopy with reduced condenser aperture may provide reasonably comparable performance when used by experienced observers [6].

Although automated urine analyzers have significantly improved the standardization of overall particle counting, they still present important limitations in the accurate identification of dysmorphic RBCs and certain types of casts. Therefore, pathological samples or those with significant abnormalities should continue to undergo manual review by appropriately trained personnel [2], 6].

Current automated urine particle analyzers are mainly based on two technological principles: flow cytometry and automated digital morphology. Flow cytometry systems classify urinary particles according to optical and fluorescence signals, including forward scatter, side scatter, fluorescence intensity, and depolarized light, often after staining with specific fluorochromes. These instruments provide robust quantitative information for common particles such as RBCs, WBCs, epithelial cells, bacteria, and some casts, and may offer indirect parameters related to erythrocyte size or scatter properties. However, these parameters are not equivalent to direct morphological recognition of dysmorphic RBCs or acanthocytes. Automated digital morphology systems, in contrast, acquire images of native urine particles either in a flow cell or in a digital cuvette and use software-assisted classification based on shape, contrast, texture, and other image features. These systems have the advantage of allowing operator review and reclassification of stored images, but their performance remains dependent on image quality, particle orientation, software libraries, and local verification.

Consequently, automated systems may be useful as screening tools, for quantitative RBC enumeration, and for flagging samples that require expert review; however, they should not currently replace visual microscopy for definitive dysmorphic RBC assessment. In samples with suspected glomerular haematuria, significant haematuria with proteinuria, pathological casts, discordant analytical findings, or analyzer flags suggesting abnormal erythrocyte morphology, manual microscopic review, preferably by phase-contrast microscopy, remains necessary. Each laboratory should establish local rules defining when automated results require microscopic confirmation and should verify the performance of the analyzer against its reference or routine manual microscopy procedure.

Regarding the quantitative assessment of erythrocyte dysmorphism, it is essential to ensure a minimum number of RBCs to allow reliable interpretation. Samples with erythrocyte counts below the upper limit of the reference interval established for a healthy population, corresponding to the 95th percentile in both men and women, defined as 20 erythrocytes/µL (approximately 2–3 erythrocytes per high-power field in uncentrifuged urine), should not be considered diagnostic [2]. This precaution is justified by the observation that a certain degree of erythrocyte dysmorphism may be present even within the range of physiological haematuria [6].

Accordingly, reporting percentages of dysmorphic RBCs should be avoided when erythrocyte counts are insufficient to support a robust evaluation. It is advisable for each laboratory to establish an internal minimum threshold of evaluable erythrocytes for reporting purposes. In the absence of such a threshold, an interpretative comment may be included, such as: “*Sample with a low number of erythrocytes, not suitable for a reliable assessment of erythrocyte dysmorphism*”.

### Integration with other urinalysis parameters

Dysmorphic RBC counts should not be interpreted in isolation, but rather in conjunction with the patient’s analytical, morphological, and clinical findings. Their diagnostic value increases when associated with albuminuria or proteinuria, reinforcing the suspicion of a glomerular origin, particularly when pathological casts, especially erythrocyte or granular casts, are also present. Interpretation is further strengthened when integrated with clinical and biochemical data such as hypertension, impaired renal function, edema, nephritic syndrome, or immunological findings suggestive of renal disease. Conversely, the predominance of isomorphic RBCs, the presence of clots, colicky pain, significant bacteriuria, atypical urothelial cells, or a relevant urological history suggests a non-glomerular or mixed origin. Accordingly, erythrocyte dysmorphism should always be evaluated within the context of the overall diagnostic framework.

## Cut-off values for dysmorphic RBCs and recommendations for laboratory reporting

There is considerable controversy regarding the optimal cut-off for the percentage of dysmorphic erythrocytes that should be considered indicative of a glomerular origin of haematuria. In this context, it is important to emphasize that the lower (i.e., less stringent) the threshold used to define glomerular disease based on the proportion of dysmorphic RBCs or acanthocytes, the lower the diagnostic specificity. This suggests that certain morphological alterations consistent with erythrocyte dysmorphism may be observed, in small proportions, even in the absence of overt glomerular disease.

According to the European urinalysis guidelines [2], the presence of ≥40 % dysmorphic RBCs, ≥2 % acanthocytes, or erythrocyte casts is suggestive of dysmorphic haematuria. Furthermore, the likelihood of a glomerular origin increases when the percentage of dysmorphic erythrocytes reaches or exceeds 80 %, or when acanthocytes account for at least 5 % of the total erythrocyte count (Table 2).

**Table 2:** Cut-off values for the clinical orientation of haematuria.

Probability	Parameter	Cut-off	Sensitivity, %	Specificity, %
Possible	Dysmorphic erythrocytes	40 %	60	50
	Acanthocytes	2 %	60	80
Probable	Dysmorphic erythrocytes	80 %	40	70
	Acanthocytes	5 %	40	90

From a clinical interpretation perspective, acanthocytes should be given greater diagnostic weight than the overall percentage of dysmorphic RBCs, particularly in borderline cases. This is because acanthocytes show higher specificity for glomerular haematuria and may therefore help confirm glomerular origin while reducing false-positive interpretation. Accordingly, when total dysmorphic RBCs and acanthocytes provide discordant or borderline information, the presence and proportion of acanthocytes should be explicitly highlighted in the laboratory report [4], 5], 7].

## Recommendations for laboratory reporting

Laboratory reports should be clear, cautious, and clinically meaningful. It is advisable to avoid overly categorical statements and instead provide an interpretative, guidance-oriented approach.

Table 3 summarizes examples of interpretative comments applicable to dysmorphic RBCs assessment in different clinical scenarios.

**Table 3:** Interpretative comments in different clinical scenarios.

Clinical scenario	Interpretative comment
Sample with low erythrocyte count (<20 erythrocytes/µL)	Assessment of dysmorphic RBCs is not appropriate, as the sample contains an insufficient number of erythrocytes.
Sample with evidence of contamination or improper collection	Sample shows evidence of contamination and is not suitable for reliable assessment of erythrocyte dysmorphism.
Aged sample or delayed processing	Delayed analysis may induce degenerative changes in urinary erythrocytes and limit the reliability of morphological assessment. Submission of a new sample is recommended.
Haematuria with clots	The presence of clots suggests a predominantly non-glomerular or urological origin of haematuria.
Haematuria with colicky pain	Clinical findings are compatible with a possible urological origin, such as urolithiasis. Dysmorphic RBCs assessment has low diagnostic yield in this context.
Haematuria with significant bacteriuria or confirmed urinary tract infection	In the presence of confirmed urinary tract infection, haematuria is usually interpreted in relation to the infectious process. Dysmorphic RBCs assessment has low diagnostic yield in this setting.
Overt macroscopic haematuria	Dysmorphic RBCs assessment has low diagnostic yield in the context of overt macroscopic haematuria.
Sample obtained after intense physical exercise, trauma, urological instrumentation, or recent catheterization	Haematuria should be interpreted with caution, as intense physical activity, trauma, urological instrumentation, or recent catheterization may cause transient haematuria and affect the diagnostic performance of erythrocyte dysmorphism.
Persistent microhaematuria confirmed in repeated samples, without proteinuria, preserved glomerular filtration rate, and predominance of dysmorphic erythrocytes	Urinary sediment shows X% dysmorphic erythrocytes (X% acanthocytes), a finding suggestive of glomerular haematuria. Preserved glomerular filtration and absence of significant proteinuria suggest an early or mild form, with conditions such as IgA nephropathy, glomerular basement membrane abnormalities, or persistent glomerular haematuria with potentially benign course being particularly relevant. Follow-up is recommended, as the absence of proteinuria does not exclude subclinical glomerular disease.
Microhaematuria with proteinuria and ≥40 % dysmorphic erythrocytes and/or ≥2 % acanthocytes	Findings compatible with haematuria of possible glomerular origin.
Microhaematuria with proteinuria and ≥80 % dysmorphic erythrocytes and/or ≥5 % acanthocytes	Findings compatible with haematuria of probable glomerular origin.

## Proposed algorithm

**Figure 2:**
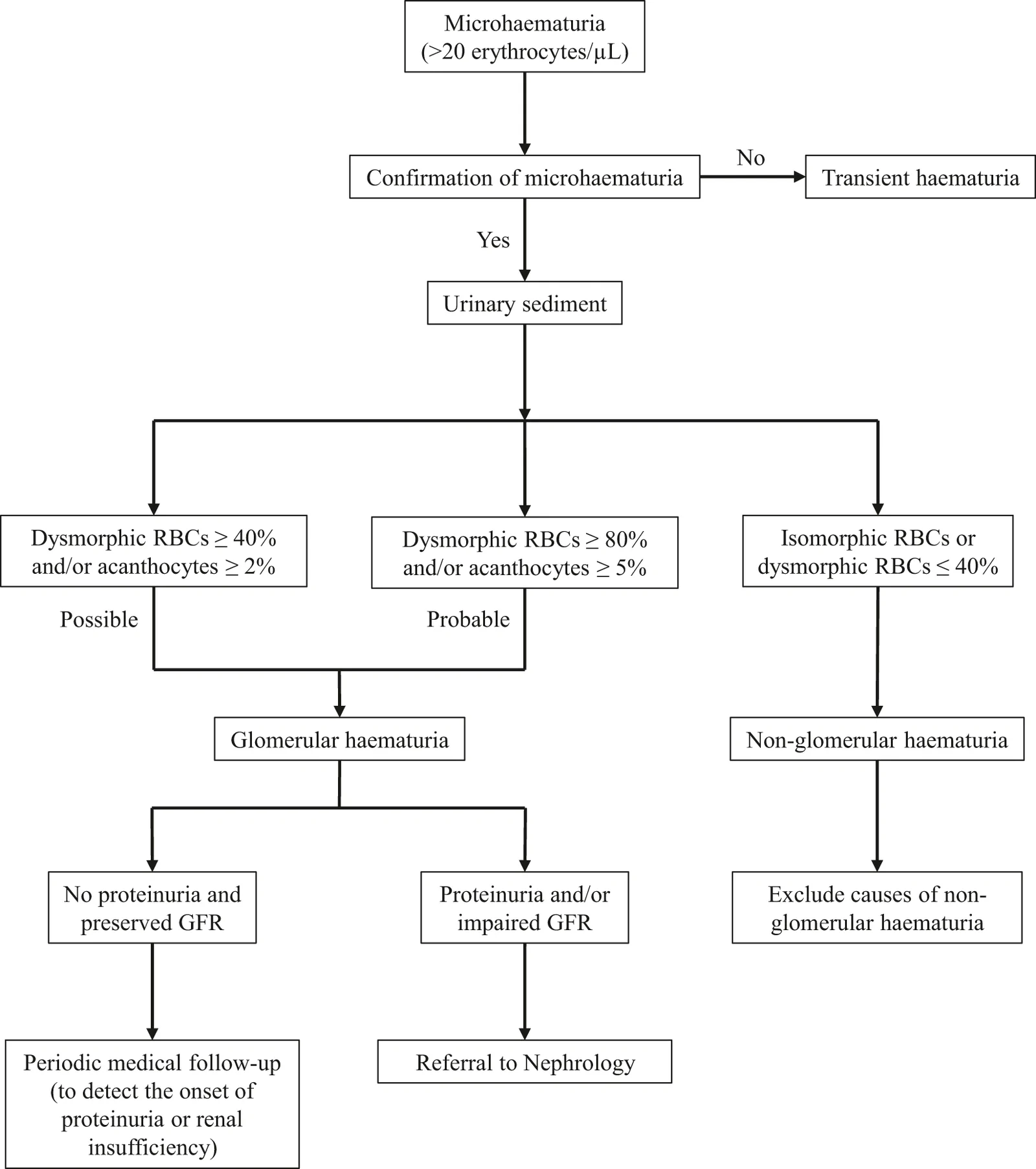
Diagnostic algorithm for the use of dysmorphic RBC counts in the evaluation of haematuria.

From a clinical perspective, dysmorphic RBC counting should be integrated sequentially into the overall evaluation of haematuria. First, the presence of haematuria must be confirmed using urine dipstick testing and/or urinary sediment analysis. Once confirmed, potential preanalytical interferences and transient causes that may bias interpretation should be excluded, including menstruation, intense physical exercise, urinary tract infection, urolithiasis, or recent urological instrumentation. Subsequently, the evaluation should be completed through an integrated assessment of proteinuria or albuminuria, renal function, and other urinary sediment findings. Within this framework, dysmorphic RBC assessment should be selectively indicated, particularly in patients with suspected glomerular haematuria or persistent haematuria of unknown origin. This diagnostic approach can be summarized in a sequential algorithm (Figure 2).

The interpretation of results should always be performed within the context of the full set of available clinical and analytical data, including comprehensive urinalysis. Based on this integrated assessment, clinical referral can be appropriately guided: a pattern suggestive of glomerular origin supports nephrology evaluation, whereas findings consistent with a non-glomerular or mixed origin require careful clinicopathological correlation and, depending on the context, consideration of urological investigation.

## Conclusions

The assessment of dysmorphic red blood cells remains a valuable component of advanced urinalysis, particularly in the evaluation of haematuria of suspected glomerular origin. However, its diagnostic utility is highly dependent on preanalytical sample quality, the analytical methodology employed, and the observer’s expertise, as well as on its appropriate integration with other laboratory findings and the clinical context.

Rather than a standalone or universally applicable test, dysmorphic RBC evaluation should be considered a specialized, technically demanding tool that is selectively indicated. When applied and interpreted appropriately, it can refine diagnostic orientation, support the identification of patients who may benefit from nephrological evaluation, and help avoid unnecessary investigations.

In this framework, proper standardization is essential to maximize its clinical value, reinforcing the role of the clinical laboratory as a key contributor in the initial stratification and management of haematuria.

## References

[1] Ingelfinger JR (2021). Hematuria in adults. N Engl J Med.

[2] Kouri TT, Hofmann W, Falbo R, Oyaert M, Schubert S, Gertsen JB (2024). European Federation of Clinical Chemistry and Laboratory Medicine (EFLM). The EFLM European Urinalysis Guideline 2023. Clin Chem Lab Med.

[3] Cohen RA, Brown RS (2003). Clinical practice. Microscopic hematuria. N Engl J Med.

[4] Saha MK, Massicotte-Azarniouch D, Reynolds ML, Mottl AK, Falk RJ, Jennette JC (2022). Glomerular hematuria and the utility of urine microscopy: a review. Am J Kidney Dis.

[5] Fogazzi GB (2010). The urinary sediment: an integrated view.

[6] Cavanaugh C, Perazella MA (2019). Urine sediment examination in the diagnosis and management of kidney disease: core curriculum 2019. Am J Kidney Dis.

[7] Köhler H, Wandel E, Brunck B (1991). Acanthocyturia – a characteristic marker for glomerular bleeding. Kidney Int.

